# Diagnosis of Compound Fault Using Sparsity Promoted-Based Sparse Component Analysis

**DOI:** 10.3390/s17061307

**Published:** 2017-06-06

**Authors:** Yansong Hao, Liuyang Song, Yanliang Ke, Huaqing Wang, Peng Chen

**Affiliations:** 1 College of Mechanical & Electrical Engineering, Beijing University of Chemical Technology, Chao Yang District, Beijing 100029, China; hys_buct@163.com (Y.H.); 18649880645@163.com (Y.K.); 2 Graduate School of Bioresources, Mie University, 1577 Kurimamachiya-cho, Tsu, Mie 514-8507, Japan; chen@bio.mie-u.ac.jp

**Keywords:** rotating machinery, compound fault diagnosis, wavelet modulus maxima, sparse component analysis

## Abstract

Compound faults often occur in rotating machinery, which increases the difficulty of fault diagnosis. In this case, blind source separation, which usually includes independent component analysis (ICA) and sparse component analysis (SCA), was proposed to separate mixed signals. SCA, which is based on the sparsity of target signals, was developed to sever the compound faults and effectively diagnose the fault due to its advantage over ICA in underdetermined conditions. However, there is an issue regarding the vibration signals, which are inadequately sparse, and it is difficult to represent them in a sparse way. Accordingly, to overcome the above-mentioned problem, a sparsity-promoted approach named wavelet modulus maxima is applied to obtain the sparse observation signal. Then, the potential function is utilized to estimate the number of source signals and the mixed matrix based on the sparse signal. Finally, the separation of the source signals can be achieved according to the shortest path method. To validate the effectiveness of the proposed method, the simulated signals and vibration signals measured from faulty roller bearings are used. The faults that occur in a roller bearing are the outer-race flaw, the inner-race flaw and the rolling element flaw. The results show that the fault features acquired using the proposed approach are evidently close to the theoretical values. For instance, the inner-race feature frequency 101.3 Hz is very similar to the theoretical calculation 101 Hz. Therefore, it is effective to achieve the separation of compound faults utilizing the suggest method, even in underdetermined cases. In addition, a comparison is applied to prove that the proposed method outperforms the traditional SCA method when the vibration signals are inadequate.

## 1. Introduction

Rotating machinery, such as roller bearings, is commonly used in industrial settings [1,2]. The fault diagnosis of rotating machinery is very important to guarantee that the whole machinery system works under normal conditions [3,4]. However, fault diagnosis is complicated because of high frequency of compound faults [5,6]. To effectively separate the compound faults, blind source separation (BSS) method was proposed [7,8]. Among BSS methods, independent component analysis (ICA) and sparse component analysis (SCA) are the most widely used algorithms. They are effective in separating the source signals from the mixed observations [9,10]. ICA is based on the statistical independence and SCA is based on the sparsity of the source signals.

In the case of compound faults, problems with equal numbers of observations and sources are called determined problems. If when the number of observations is greater than the number of sources, then they are defined as overdetermined problems. For some determined problems and overdetermined problems, the ICA methods perform well on separation [11,12]. However, the actual signals are usually collected by fewer sensors than the total number of sensors available because some sources are affected by environmental conditions, which makes them underdetermined problems, as the number of observations is less than that of sources. To solve this problem, certain classical algorithms, such as ensemble empirical mode decomposition (EEMD), were used to decompose a single channel signals into multiple signals [13,14]. However, the number of decomposition channels is often more than the number of source signals, which makes the separation of source signals contain false source signals. Moreover, the EEMD-based ICA method takes a long time to carry out. In addition, the ICA BSS has the precondition that the source signals satisfy non-Gaussian distribution and are independent of each other [15,16]. All of this means that the above methods are not applicable in dealing with compound faults under the underdetermined conditions.

With an aim to solve the above-mentioned problems, sparse component analysis was gradually developed. It makes full use of signal sparse characteristics to realize the source signal separation. It can effectively separate sparse source signals when ICA does not perform well. The concept of the sparsity model was first proposed by Olshausen [17], and later the concept of SCA was raised by Lewicki [18,19]. The concept that SCA can completely reconstruct the source signal in BSS was put forward by Georgiev et al. [20,21]. In addition, Bofill et al. presented the method of estimating the mixed matrix by the potential function (PF) and successfully separated the mixed sound signals [22]. 

To separate the observed signals using the SCA method, the sparsity of the target signals is one of the most significant conditions that must be satisfied. Source signals, such as sine signal and cosine signal, can be represented sparsely by Fourier Transform. However, in the case of vibration signals, it is very difficult to disperse signals in a sparse way using traditional methods, such as Fourier Transform and wavelet transform. Therefore, to represent the vibration signals sparsely, a sparsity-promoted method using the wavelet modulus maxima is proposed in this paper. First, the wavelet modulus maxima are used to achieve the objective of thinning the mixed signals. Then, the number of mixed signals and the mixed matrix are estimated by PF based on the sparsity of the observed signals. Finally, the shortest path method is used to separate the source signals. In addition, a comparison is made with the traditional envelope spectrum method and the SCA method in order to verify the effectiveness of the proposed method.

The rest of the paper is organized as follows. The basic theories of sparse component analysis and wavelet modulus maxima are introduced in Section 2. Section 3 presents the detailed steps to separate the compound faults based on the proposed method. Section 4 mainly discusses the research on simulation signals and experimental signals. Lastly, the conclusions are drawn in Section 5.

## 2. Basic Theory

### 2.1. Sparse Component Analysis

Sparse component analysis algorithms require that signal is sparse enough. The so-called sparse signal refers to the signal that most of the time is zero (or close to zero), while it is bigger than zero a few times. The probability density function distribution diagram shows a clear wave crest and smooth descent. If the source signal is a sparse signal, the zero value (or close to zero) will be far more frequent than the nonzero value (or larger than zero), and only one source signal can be dominant at most times [23,24]. 

The model can be expressed as:

(1) $$X_{m\times T}=A_{m\times n}S_{n\times T}\left(m\leq n\right),$$

where $X=\left[X_{1},X_{1},\cdots ,X_{T}\right]$ represents the observation signal; *A* represents a mixed matrix; and *S* represents the source signal.

The PF method utilizes a type of algorithm based on sparse clustering data to calculate the source number and mixed form of the underdetermined blind signals [22]. The PF method assumes that the number of mixed signals is two, let $l_{t}=\sqrt{x_{1}^{2}(t)+x_{2}^{2}(t)}$ and $\theta (t)=\tan^{-1}(x_{2}(t)/x_{1}(t))$, and PFs Φ are defined as shown in the following Equation:

(2) $$\Phi (\theta ,\lambda )=\sum_{t}l_{t}\varphi (\lambda (\theta -\theta (t)))$$

where the basis function φ around a triangular function of the local angle α is based on Equation (3), λ is used to adjust the desired angular width or resolution of the local contributions, which is the actual angular difference between an arbitrary direction and $\theta_{t}$ in the polar coordinates, and $l_{t}$ is a weight to put more emphasis on the more reliable data:

(3) $$\varphi (\alpha )=\left\{ \begin{array}{ll} 1-\frac{\alpha }{\pi /4} & \left|\alpha \right|<\pi /4\\ 0 & \mathrm{elsewhere} \end{array}\right..$$

The PF Φ shows the distribution of the sampling data in the cluster center, if the column vector of the mixed matrix A is just in the actual sampling data, the PF of the column vector will be large. When the number of local maxima is exactly equal to the number of vibration sources, the corresponding column vector is an approximate solution about the mixed matrix $\hat{A}$. Thus, the number of the vibration sources and the mixed matrix $\hat{A}$ are obtained.

When the mixing matrix A is known, the solution is not unique, as the system in Equation (4) is underdetermined. The usual approach of sparse BSS consists of finding the solution that minimizes the $l_{1}$ norm. In this case, the optimal representation of the data point:

(4) $$x\left(t\right)=\sum_{i=1}^{n}a_{i}s_{i}\left(t\right),\;\mathrm{and}\;\sum_{i=1}^{n}\left|s_{i}\left(t\right)\right|=\min.$$

The shortest path method is to find the shortest path from the origin to x(t), and the smallest sum of the vector length is the solution in all combinations by decomposing the observed signals. The shortest path decomposition involves the following steps [25]:

(1) Extract any m column vector from the estimated mixed matrix $\hat{A}$ to compose the m×m dimensional matrix termed $\overset{*}{A}$ and get the $C_{n}^{m}$ matrix;
(2) Calculate the inverse matrix ${\overset{*}{A_{i}}}^{-1}$ of each matrix, $i=1,\cdots ,C_{n}^{m}$;
(3) Decompose the observed signal vector $x^{t}$ according to the direction of the base vector, which is every column vector in the matrix, and the length of each vector is obtained. For any time *t*, the observed signal is formed by a linear combination of the m sparse source signals, so that the source signal is decided by the next Equation:
  (5) $$\{\begin{array}{l} s_{r}\left(t\right)={\overset{*}{A_{i}}}^{-1}\times x\left(t\right),\sum_{r}\left|s_{r}\left(t\right)\right|=\min,r\in \left(1,\cdots ,n\right)\\ s_{i}\left(t\right)=0,\;i\in \left(1,\cdots ,n\right),i\neq r. \end{array}$$

Therefore, the source signal can be calculated for all times according to the Equation, which decomposes the source signal estimation into *T* linear programming problems.

### 2.2. Wavelet Modulus Maxima

The method of wavelet analysis is the extension of the Fourier analysis method, which is the local transformation of space (time) and frequency, so it can effectively extract the information from the signal and solve many difficult problems that cannot be solved by Fourier Transform. 

Discretization of the continuous wavelet is the discretization of the continuous scale parameter *s* and translation parameter τ. Usually, the continuous scale parameter s and the translation parameters τ in wavelet transform are taken as ${s=s}_{0}^{j}$, $\tau =\tau_{0}^{j}$, j∈Z. Accordingly, the wavelet function can be expressed as [26,27]:

(6) $$\psi_{\;\tau_{0},s_{0}}\left(t\right)=\frac{1}{\sqrt{s_{0}^{j}}}\psi \left(\frac{t-\tau_{0}^{j}}{s_{0}^{j}}\right),$$

and the corresponding discrete wavelet transform can be expressed as:

(7) $$dwtX=\frac{1}{\sqrt{\left|s_{0}^{j}\right|}}\int_{-\infty }^{+\infty }x\left(t\right)\psi^{*}\left(\frac{t-\tau_{0}^{j}}{s_{0}^{j}}\right)dt.$$

If the wavelet function for the wavelet transform ψ is the first derivative or first derivative of a low-pass function, the results of the wavelet transform will reflect the extreme point of a signal singularity or turning point. The definition of a signal singularity can be expressed as follows.

The function f(x) has a Lipchitz index α at the point $x_{0}$, if and only if there is a constant K>0, such that ∀x∈R, and

(8) $$\left|f\left(x\right)-f\left(x_{0}\right)\right|\leq K{\left|x-x_{0}\right|}^{\alpha }.$$

It can be seen from Equation (8) that the local Lipchitz index α of the function f(x) in the point $x_{0}$ depicts the singular class type of the point. Specifically, α>1 represents f(x), which is differentiable at the point $x_{0}$. 0<α< represents f(x), which is continuous but not differentiable at the point $x_{0}$. α=0 represents f(x), which is not continuous but bounded at the point $x_{0}$, while α<0 represents f(x), and has a singularity at the point $x_{0}$, which is actually noise.

When using this local singularity of wavelet analysis, the wavelet coefficients depend on the characteristics of f(x) in the $x_{0}$ neighborhood and the size of the scale selected by the wavelet transform. In wavelet transform, the local singularity of the signal is defined as follows.

The necessary and sufficient condition for the existence of a consistent Lipchitz index α about the function f(x) in the interval [a,b] is that there exists a constant K>0, such that ∀x∈[a,b], whose wavelet transform satisfies

(9) $$\left|W_{2^{j}}f\left(x\right)\right|\leq K{\left(2^{j}\right)}^{\alpha }.$$

Taking the logarithm on both sides above and we get

(10) $$\log_{2}\left|W_{2^{j}}f\left(x\right)\right|\leq \log_{2}K+\alpha j.$$

Then, α is called the singularity index at $x_{0}$ (also known as the Lipchitz index).

The above equation gives the variation law of the logarithm of the wavelet transform coefficient with the scale j, and the variation of the modulus of the wavelet transform with the scale j at the singular point of the corresponding signal will naturally meet this rule. If the Lipchitz index α>0 in the function f(x), the modulus maxima of the function wavelet transform coefficient will increase with the scale. On the contrary, when α<0, the maximum value of the transformation modulus will decrease as the scale increases. For the case of α=0, the maximum value of the wavelet transform modulus does not change with the scale.

The extreme point signal or turning point is called the singular point, which mainly reflects the characteristics of the signal. For the actual signal, the Lipchitz index must have α>0. Then, the wavelet transform modulus maxima of the signal will increase with the scale increase. This propagation characteristic is the basic principle and basis of the signal sparse representation of the wavelet modulus maxima. Since the extreme point signal is more stable, the wavelet modulus maxima are more suitable to improve the sparsity of the signal. The steps of thinning established upon wavelet modulus maxima are as follows:

(1) Wavelet basis function is used to conduct *n* level wavelet transform to get the high-frequency wavelet coefficients at each level and obtain the wavelet modulus maxima.
(2) For each level, search for their communication points and a neighborhood on the previous level.
(3) Keep the maxima points in the neighborhood of the communication points while removing the modulus maxima from the neighborhood.

## 3. Separation Method by Sparsity Promoted-Based SCA

There are mainly two ways to solve the underdetermined BSS problem. One is decomposing the original signals into multiple channel signals, through which the underdetermined BSS issue can be transferred to determined or overdetermined problem. In this way, the EEMD-based ICA method is commonly used. The other method is SCA, which separates mixed signals based on the sparsity. However, the EEMD-based ICA method is often time-consuming and is likely to receive false source signals. In addition, the vibration signals generated by a roller bearings fault cannot be represented sparsely in the normal way. Thus, the SCA method might not perform well when the compound faults exist. To meet the requirement of sparsity, the wavelet modulus maxima are utilized to represent the vibration signals in a sparse way. 

**Figure 1:**
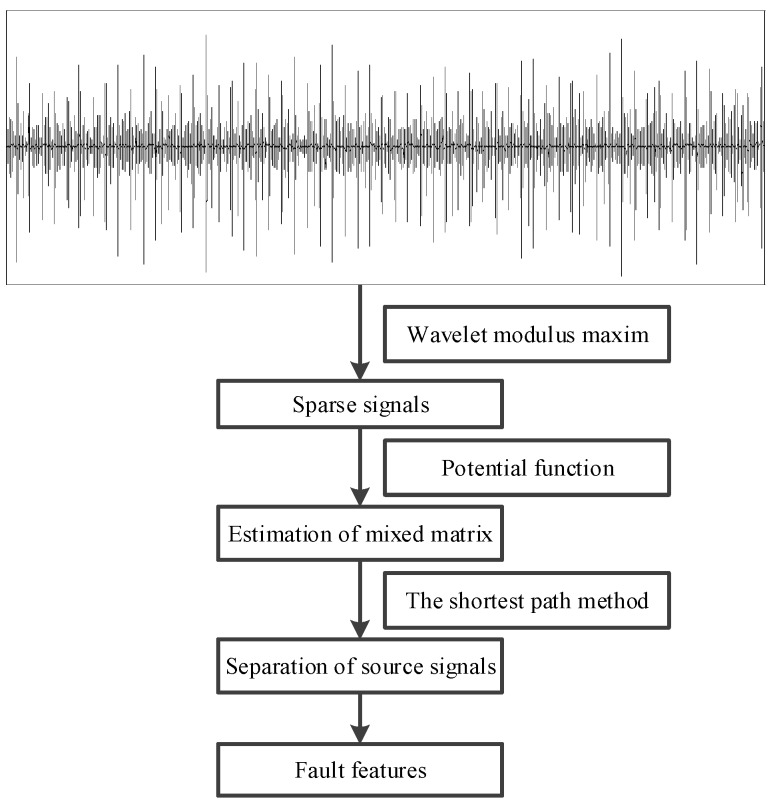
Flowchart of the proposed method.

A new method based on the wavelet modulus maxima and sparse underdetermined BSS is proposed in this paper. Several experiments on roller bearings’ faults are performed to verify the efficiency of the presented method. The results obtained by the proposed method are compared with the traditional SCA method, whose flowchart is shown in Figure 1. First, the roller bearings signals are collected by the acceleration sensor. Second, the db4 wavelet is used to perform four-level wavelet transform to get the high frequency wavelet coefficients of each level and acquire the modulus maxima. Then, the maxima points are removed out of the neighborhood of the communication points. Keep the maxima points in the neighborhood and the sparse mixed signals are obtained. The sparse signal that has the most obvious peaks and the prominent turning point in the potential function is chosen as the following experimental object. Third, the PF is used to estimate the mixed matrix and the number of sources, and the source signals were separated according to the shortest path. Lastly, the roller bearing fault features are extracted by comparing them with the theoretical characteristic frequencies of a roller bearing.

## 4. Application Cases

### 4.1. Simulation Analysis

**Figure 2:**
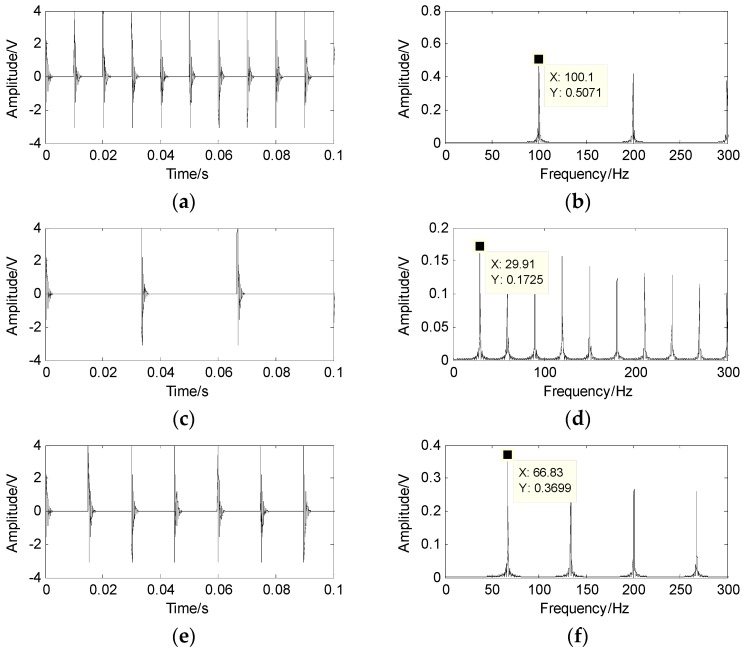
Source signal and its spectrum. (**a**) the waveform of $s_{1}$; (**b**) the spectrum of $s_{1}$; (**c**) the waveform of $s_{2}$; (**d**) the spectrum of $s_{2}$; (**e**) the waveform of $s_{3}$; (**f**) the spectrum of $s_{3}$.

To prove the applicability of the SCA algorithm in underdetermined BSS applications, three sparse source signals are generated according to Equation (11), whose characteristic frequencies are 100 Hz, 67 Hz and 30 Hz. The sampling frequency is $f_{s}=20,000$ Hz and the sampling data length is N=40,960. The spectrum of the source signal is presented in Figure 2. The three signals are randomly mixed into two observed signals $x_{1}$ and $x_{2}$ according to Equation (12), and the random mixed matrix A is expressed as $A=\left[\begin{array}{ccc} 0.3446 & 0.5827 & 0.2003\\ 0.9387 & 0.8127 & 0.9797 \end{array}\right]$,

(11) $$s(t)=y_{0}e^{-\xi \omega_{n}t}\sin\omega_{n}\sqrt{1-\xi^{2}}t,$$

(12) $$x(t)=As(t)=A{\left[s_{1}(t),s_{2}(t),s_{3}(t)\right]}^{T}.$$

**Figure 3:**
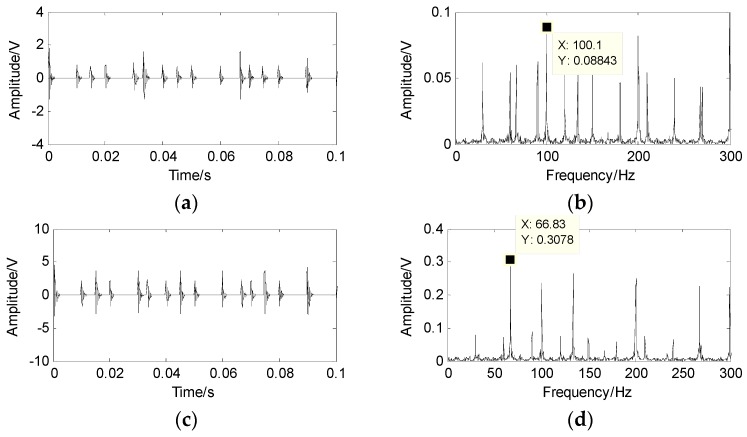
Mixed of simulated signals and their spectra. (**a**) the first mixed signal $x_{1}$; (**b**) the spectrum of $x_{1}$; (**c**) the second mixed signal $x_{2}$; (**d**) the spectrum of $x_{2}$.

Figure 3 shows the mixed signals and their spectra. The characteristic frequency cannot be obtained from the results shown in Figure 3. In this case, the wavelet modulus maxima are employed to generate the sparse signal. First, the db4 wavelet is used to perform the four-level wavelet transform to get the high-frequency wavelet coefficients of each level and acquire the modulus maxima. Second, search for their communication points on the previous level and then the maxima points are kept, as they are in the neighborhood of the communication points, while the modulus maxima are removed, as they are out of the neighborhood. Finally, the sparse signals are gained.

**Figure 4:**
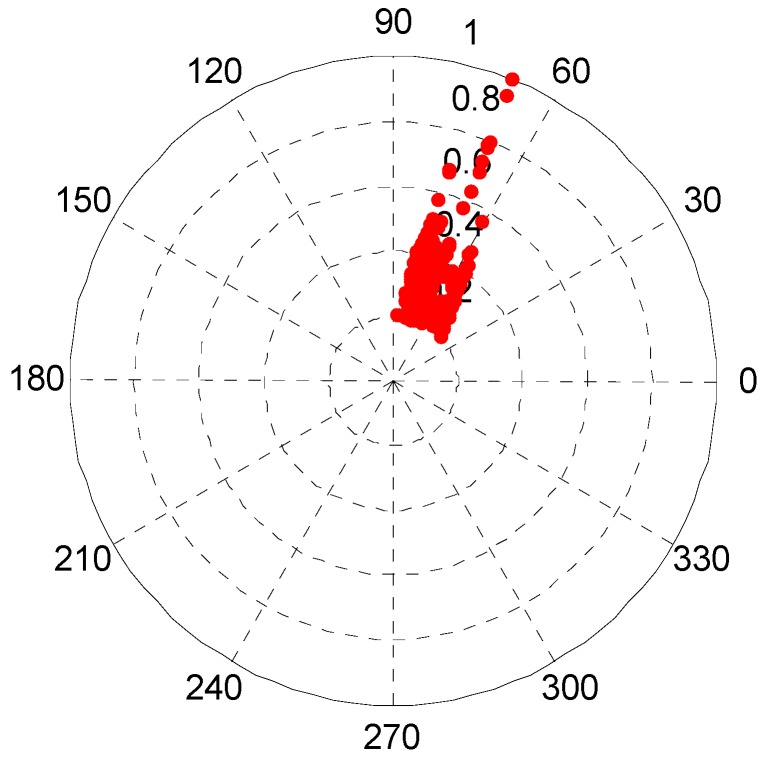
The polar plot of the scatter diagram about the mixed signals.

**Figure 5:**
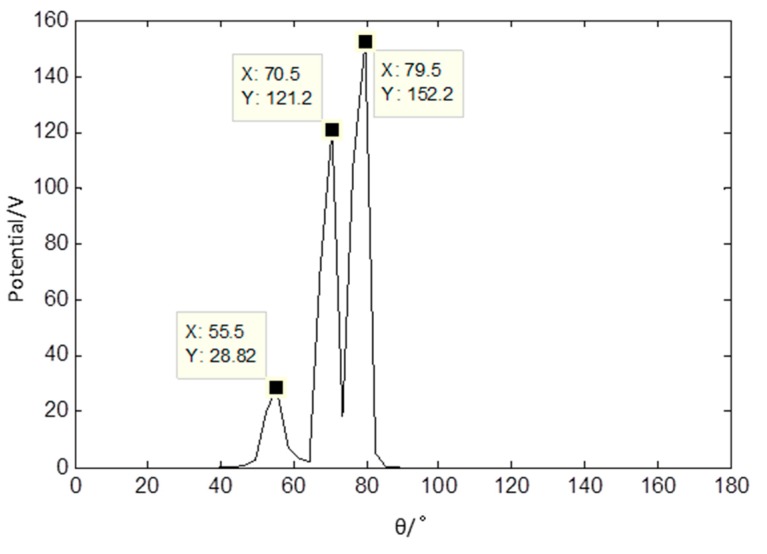
Potential function of the mixed signals.

The scatter diagram of the sparse signals is demonstrated in Figure 4, and the sparse signals PF are shown in Figure 5. It can be seen that the peaks and turning points are apparent, which means that the sparsity of signal is appropriate. The mixed signals number and the estimation of the mixed matrix $\bar{A}$ are obtained from the results shown in Figure 4 and Figure 5. The number of sources is three and the mixed column vector is ${\left[\cos\theta_{k},\sin\theta_{k}\right]}^{T}$, which is presented in $\bar{A}=\left[\begin{array}{ccc} 0.5620 & 0.3338 & 0.1822\\ 0.8241 & 0.9426 & 0.9833 \end{array}\right]$, where the estimation of the mixed matrix is very close to the mixed matrix.

**Figure 6:**
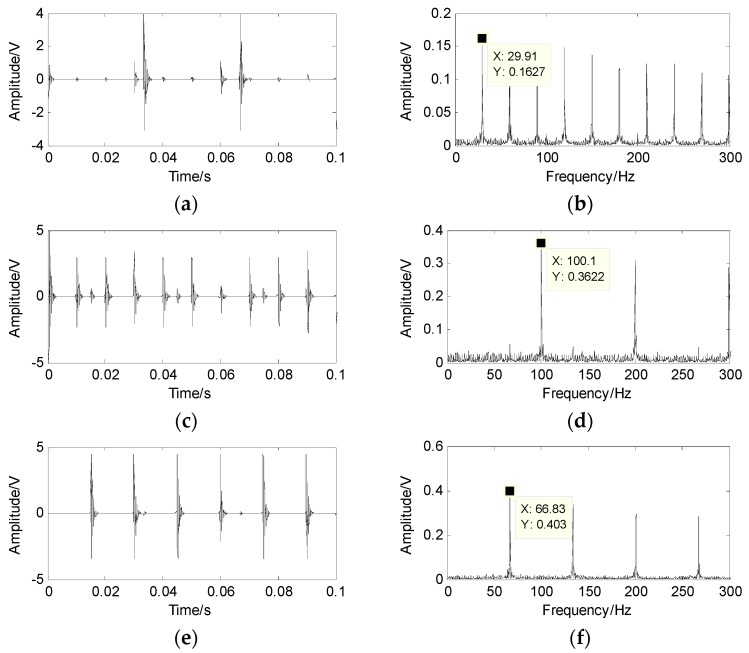
The separation of the simulated signals and their spectra. (**a**) the first separation of signal; (**b**) the spectrum of the first separation; (**c**) the second separation of signal; (**d**) the spectrum of the second separation; (**e**) the third separation of signal; (**f**) the spectrum of the third separation.

Finally, the source signal is separated based on the shortest path method and the results are shown in Figure 6, which are very close to the results displayed in Figure 2. The results reveal that the proposed method works well in underdetermined BSS.

### 4.2. Experimental Verification and Discussion

**Figure 7:**
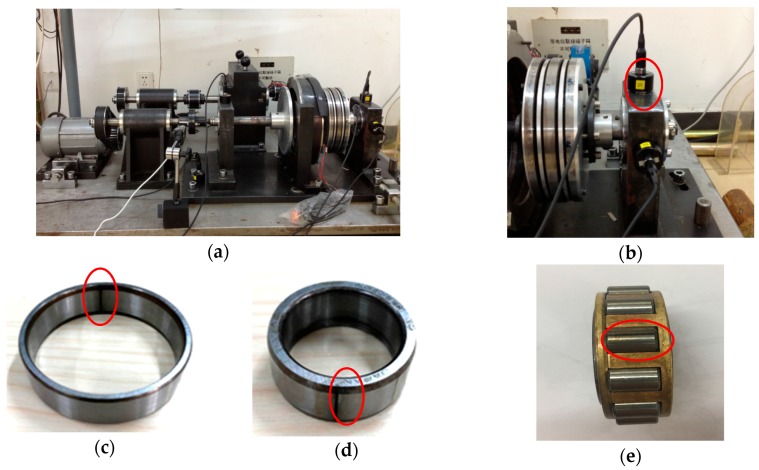
Experimental system for bearing diagnosis. (**a**) experiment table; (**b**) install location of the acceleration sensor; (**c**) outer-race and its flaw; (**d**) inner-race and its flaw; (**e**) roller and its flaw.

Aiming at verifying availability of the method proposed in this paper, the experimental system of the bearing diagnosis is used. As is shown in Figure 7a, the experimental system includes a rotating machine, the roller bearing (NTN 204) and acceleration sensors. A 1-kW ac motor is employed to drive the experimental setup and the measurements in the experimental system are made without load. The accelerometer sensors are mono-axial piezoelectricity type accelerometers (IMI 608A11) with a bandwidth from 0.5 Hz to 10 KHz and a 100 mV/g sensitivity. The vibration signals of the roller bearings faults are collected through two accelerometer sensors that are mounted on the bearing housing in the vertical and horizontal directions, as indicated in Figure 7b. In addition, the signals used in this paper are obtained by the sensor in the vertical direction due to the fault features being more obvious in this direction. The faults often occurring in a roller bearing are the outer-race flaw, the inner-race flaw and the rolling element flaw. We artificially made those flaws as shown in Figure 7c,d,e for the tests of condition diagnosis. Technical characteristics of the NTN N204 bearing are displayed in Table 1. The sizes of the flaws in roller bearings are all 0.5 × 0.15 mm (width ×depth), and the failures were artificially created using a wire-cutting machine. The inherent frequencies of roller bearing are generally distributed in the high frequency band. To fully analyse signal features, considering further research, the larger sampling frequency is chosen to acquire the more comprehensive information of machinery conditions. Thus, the sampling frequency is 100 KHz and the sampling time as 10 s. The corresponding time of vibration signals used in this study is 1 s. The vibration signals collected at 900 rpm is utilized to verify the effectiveness of the proposed method.

(13) $$f_{o}=\frac{Z}{2}(1-\frac{d}{D}\cos\alpha )f_{r},$$

(14) $$f_{i}=\frac{Z}{2}(1+\frac{d}{D}\cos\alpha )f_{r},$$

(15) $$f_{b}=\frac{D}{2d}[1-(\frac{d}{D}\cos\alpha {)}^{2}]f_{r}.$$

The fault passing frequency of each element of the roller bearings can be calculated according to Equations (13)–(15), where D is the pitch diameter, Z is the number of rollers, d is the diameter of the rollers, α is the contact angle of the rollers, and $f_{r}$ is the rotating frequency. $f_{o}$, $f_{i}$ and $f_{b}$ represent the frequency of the outer-race, inner-race and the rollers, respectively. These equations are based on the assumption of a pure rolling motion. However, in practice, some sliding motion may occur, which causes slight deviation in the characteristic frequency locations. Therefore, these equations should be regarded as approximations only. The approximate results are shown in Table 2.

**Figure 8:**
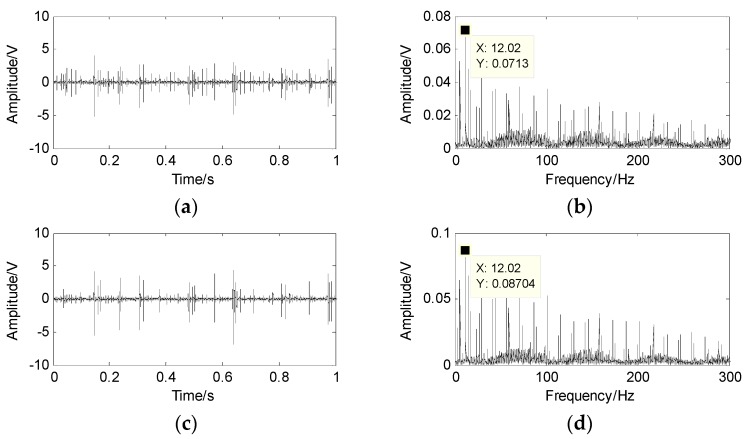
Mixed signal and its spectrum. (**a**) the first mixed signal $x_{1}$; (**b**) the spectrum of $x_{1}$; (**c**) the second mixed signal $x_{2}$; (**d**) the spectrum of $x_{2}$.

The faults of the outer-race, inner-race and the rollers are mixed into two signals $x_{1}$ and $x_{2}$ by a random matrix. The waveforms and the spectrum of the mixed signals are shown in Figure 8, where the fault characteristic frequency cannot be obtained from the envelope spectrum of the mixed signals.

**Figure 9:**
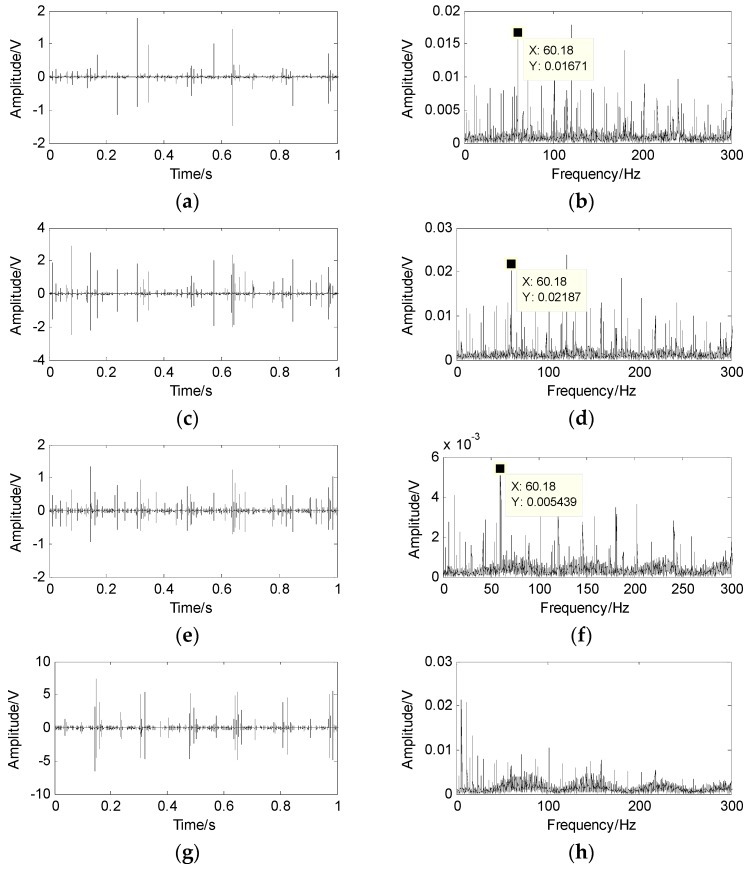
The wavelet modulus maxima of the first mixed signal and its spectrum. (**a**) wavelet modulus maxima of the first level; (**b**) the spectrum of the first level; (**c**) wavelet modulus maxima of the second level; (**d**) the spectrum of the second level; (**e**) wavelet modulus maxima of the third level; (**f**) the spectrum of the third level; (**g**) wavelet modulus maxima of the fourth level; (**h**) the spectrum of the fourth level.

**Figure 10:**
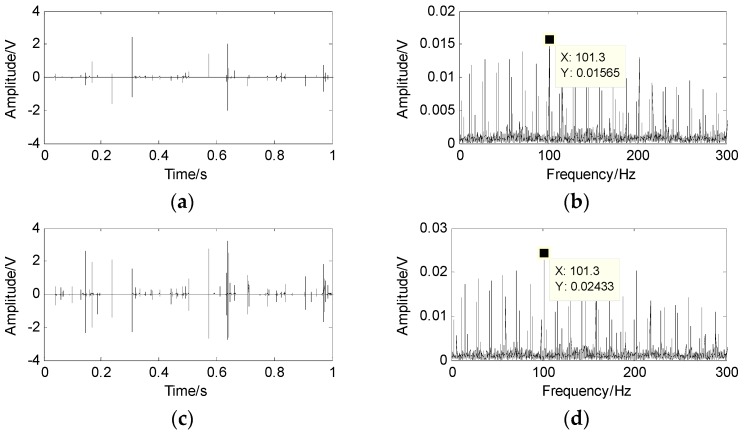

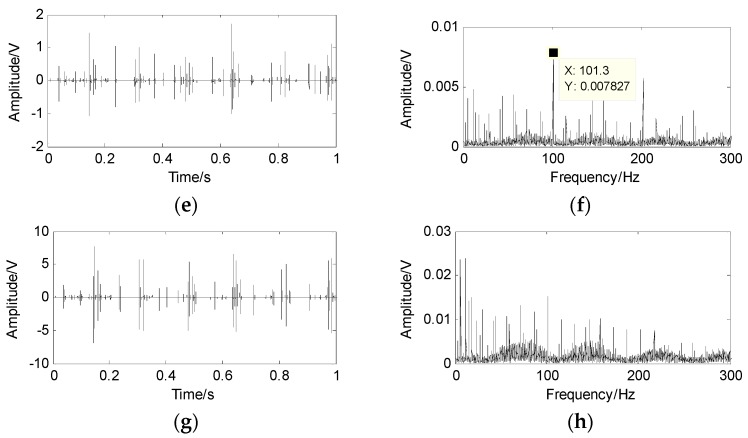
The wavelet modulus maxima of the second mixed signal and its spectrum. (**a**) wavelet modulus maxima of the first level; (**b**) the spectrum of the first level; (**c**) wavelet modulus maxima of the second level; (**d**) the spectrum of the second level; (**e**) wavelet modulus maxima of the third level; (**f**) the spectrum of the third level; (**g**) wavelet modulus maxima of the fourth level; (**h**) the spectrum of the fourth level.

The wavelet modulus maxima are used to thin the mixed signals to achieve the linear clustering. First, the db4 wavelet is used to implement the four-level wavelet transform to acquire the high frequency wavelet coefficients of each level and obtain the wavelet modulus maxima. Second, their communication points on the previous level are located and then the maxima points are kept because they are in the neighborhood of the communication points, whereas the modulus maxima are removed because they are far from the communication points. The wavelet modulus maxima and their spectra are displayed in Figure 9 and Figure 10. Only the 60.18 Hz and 101.3 Hz can be intangibly recognized from the envelope spectrum and the rollers defect cannot be diagnosed.

**Figure 11:**
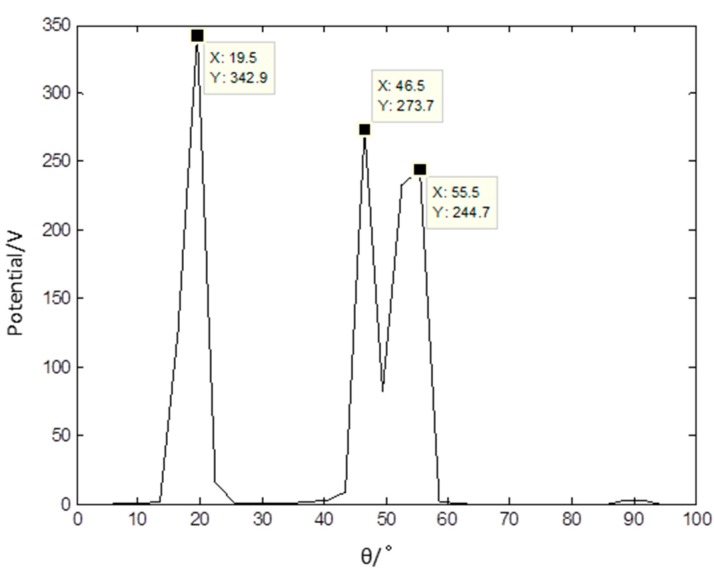
Potential function of sparse signals based on the proposed method.

The wavelet modulus maxima in the fourth level of each mixed signal are used as sparse signal for it has the appropriate sparsity. The PF of the sparse signal is presented in Figure 11 and it shows that there are three distinct peaks. The data in Figure 11 indicate that the linear clustering effect is apparent and the estimated number of source signals gained is three. The estimation of the mixed matrix could be obtained based on the PF.

**Figure 12:**
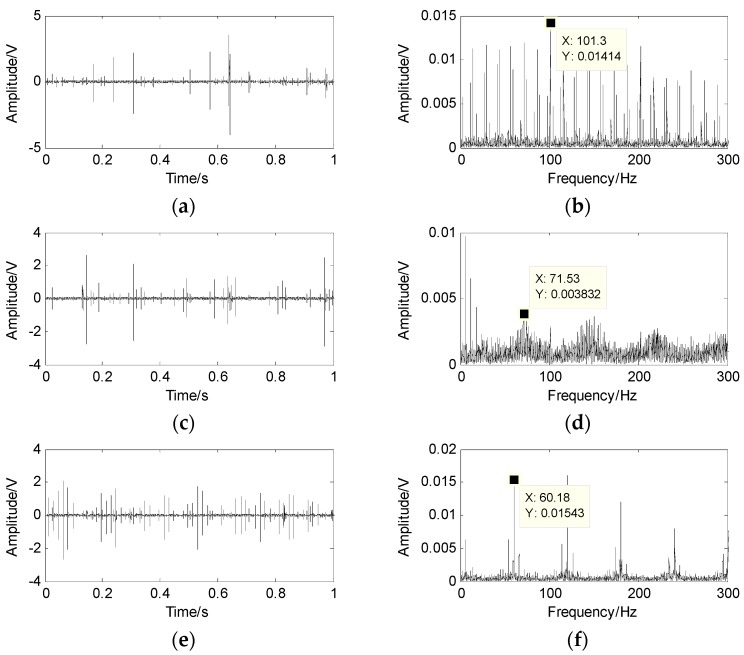
The separation of source signal and its spectrum by the proposed method. (**a**) waveform of inner-race defect; (**b**) spectrum of inner-race defect; (**c**) waveform of roller defect; (**d**) spectrum of roller defect; (**e**) waveform of outer-race defect; (**f**) spectrum of outer-race defect.

Finally, the source signal is obtained on account of the shortest path method and the results are demonstrated in Figure 12, which show the separation of the source signal and its spectrum. The frequency 60.18 Hz is evidently close to the theoretical calculation $f_{o}$ in Figure 12f. Similarly, the frequencies 101.3 Hz and 71.53 Hz are also very close to the theoretical calculation $f_{i}$ and $f_{b}$. The results indicate that the outer-race defect, inner-race defect and roller defect of the roller bearings can be separated from the compound faults. The suggested method performs better in underdetermined BSS.

### 4.3. Comparison with the Traditional SCA

To confirm that the sparsity is the key part of the SCA method and the proposed method effectively improves the sparsity of the signal, the fault diagnosis method based on the traditional SCA is used. First, the sparse signal is obtained according to the wavelet transform, the number of mixed signals and the mixed matrix are estimated by the PF according to the sparse signals.

**Figure 13:**
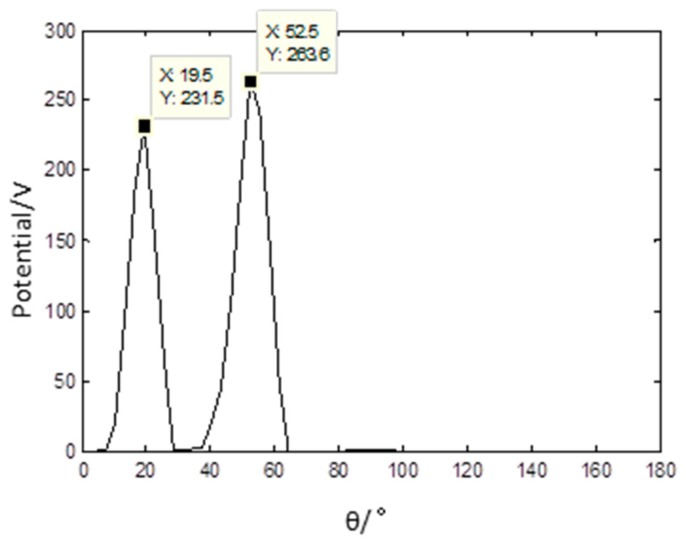
Potential function of sparse signals based on wavelet transform.

The PF is shown in Figure 13, and the number of source signals is only 2. Then, the shortest path method is used to separate the source signal.

**Figure 14:**
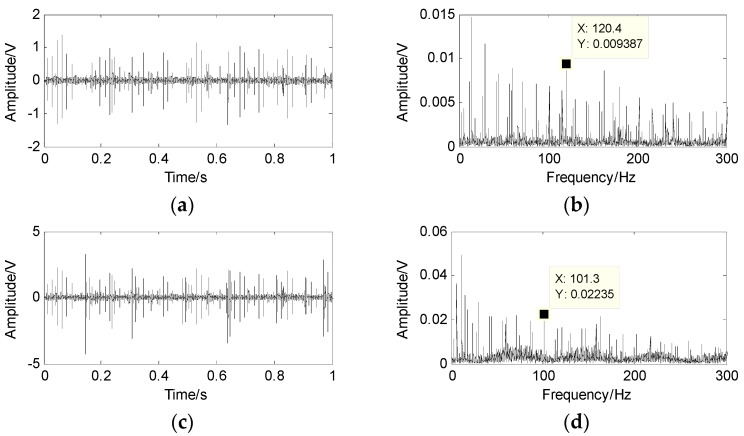
The separated source signal and its spectrum by traditional SCA method. (**a**) the first separation of signal; (**b**) spectrum of the first separation signal; (**c**) the second separation of signal; (**d**) the spectrum of second separation signal.

The waveforms and spectrum of the separated source signal are shown in Figure 14, where the fault feature of the inner-race is 101.38 Hz, but the characteristics of the outer-race and the roller are not clearly extracted. By contrast, the fault of the outer-race, inner-race and roller can be separated using the proposed method, and the fault features of the signals are very clear.

## 5. Conclusions

A promising sparse component analysis method based on the wavelet modulus maxima is proposed in this paper to extract compound fault features of roller bearings for underdetermined BSS. To overcome the insufficient sparsity when the SCA is performed, wavelet modulus maxima is used to promote the sparsity of the vibration signals. Then, the PF is employed to estimate the mixed matrix. When the mixed matrix is known, the smallest sum of the vector length is the solution in all combinations by decomposing the observed signal according to the shortest path method, and the separation of the compound faults are obtained. To verify the validity of the proposed method, the simulated signals and vibration signals of faulty roller bearings are used. The results indicate that the fault features of roller bearing with out-race defects, inner-race defects and rollers are basically the same as the theoretical value. For example, the inner-race feature frequency 101.3 Hz is very similar to the theoretical calculation 101 Hz. Therefore, compound faults can be separated based on the presented method. In addition, compared with the traditional SCA method, the experimental results showed that the proposed method successfully thins the mixed signal and extracts compound fault features. In this approach, the time for separating the source signal needs to be further reduced. Future work will focus on the point of the method.

## Figures and Tables

**Table 1:** Structure parameters of NTN N204 bearing**.**

Number of Rollers	External Diameter (mm)	Inner Diameter (mm)	Width (mm)
10	47	20	14

**Table 2:** Fault characteristic frequency of the roller bearings at 900 rpm.

Fault Location	Fault Characteristic Frequency
Outer race	60 Hz
Inner race	101 Hz
Roller	72 Hz
